# Prevalence of oral frailty in community-dwelling older adults: a systematic review and meta-analysis

**DOI:** 10.3389/fpubh.2025.1423387

**Published:** 2025-05-01

**Authors:** Jun-kai Dou, Huan Liu, Yan Mei, Song Wang, Ying Zhang, Shao-hua Zhao, Xue-zhi Shi

**Affiliations:** ^1^Department of Rehabilitation Medicine, Lu'an People's Hospital, Lu'an, Anhui, China; ^2^Department of Hemodialysis, The First Affiliated Hospital of Wannan Medical College (Yijishan Hospital of Wannan Medical College), Wuhu, Anhui, China; ^3^Department of Hemodialysis, Lu'an People's Hospital, Lu'an, Anhui, China; ^4^Department of Intensive Care Unit, Lu'an People's Hospital, Lu'an, Anhui, China; ^5^Department of Nephrology, Lu'an People's Hospital, Lu'an, Anhui, China; ^6^Department of Operating Room, Lu'an People's Hospital, Lu'an, Anhui, China; ^7^Department of Nursing, Lu'an People's Hospital, Lu'an, Anhui, China

**Keywords:** oral frailty, older adults, prevalence, community-dwelling, meta-analysis

## Abstract

**Background:**

Older adults are vulnerable to oral frailty due to factors such as age, education level, physical condition, and limited access to medical resources. Given that oral frailty can lead to adverse outcomes and is often overlooked by policymakers and health professionals, it is important to understand the current state of oral frailty among community-dwelling older adults.

**Design:**

Systematic review and meta-analysis.

**Methods:**

Two researchers independently conducted searches in seven databases, extracted data, and assessed the quality of eligible studies. Data from cross-sectional studies or cohort studies with a clear definition of oral frailty. Stata 14.0 was utilized to evaluate the overall prevalence of oral frailty, while Cochrane's *Q, I*^2^statistics were employed to assess statistical heterogeneity.

**Results:**

A total of 15 studies were ultimately included in this analysis. The pooled prevalence of oral frailty among community-dwelling older adults was 32% (95% CI: 24%−40%, *I*^2^ = 98.9%, *P* < 0.001). By country, the prevalence was 53% (95% CI: 42%−65%) in China and 22% (95% CI: 19%−39%) in Japan. The incidence of oral frailty was 29% (95% CI: 18%−39%) among those aged 74 and over and 26% (95% CI: 16%−36%) among those under 74. The prevalence of oral frailty was 46% (95% CI: 31%−60%) as assessed by the OFI-8 scale, 18% (95% CI: 14%−22%) using the OF-6 scale, and 37% (95% CI: 34%−39%) with the OFI-5 scale. The rates of oral frailty reported before 2021 and between 2022–2024 were 17% (95% CI: 13%−21%) and 42% (95% CI: 31%−53%), respectively. The rate of oral frailty was 39% (95% CI: 23%−54%) for sample sizes ≤ 500, and 25% (95% CI: 16%−33%) for sample sizes >500. Univariate meta-regression analysis revealed that country, measurement method, and publication year might be sources of heterogeneity. Funnel plot analysis and Egger's test showed no significant publication bias among the eligible studies.

**Conclusion:**

Our study found that oral frailty affects more than one in three older adults living in the community. This highlights the importance for policymakers and health professionals to screen early and implement effective measures to prevent oral frailty among older adults residing in community settings.

**Systematic review registration:**

https://www.crd.york.ac.uk/prospero/#searchadvanced, identifier: CRD42024527800.

## Introduction

As the global population ages, an increasing number of older individuals will experience age-related chronic diseases and geriatric syndromes, which have negative impacts on their families and public health care systems. Among those geriatric syndromes, oral frailty, as a novel concept, has attracted more attention worldwide in recent years.

Oral frailty, an age-related syndrome, is defined as a series of phenomena and processes in which a decline in oral health conditions, such as tooth loss, chewing disability, and swallowing disorders, results in physical and mental disorders (1). To date, there are two frequently used assessment tools: Oral Frailty-6 (OF-6) and Oral Frailty Index-8 (OFI-8). OF-6 developed by Tanaka et al. (2) defined co-existing poor oral status in ≥3 of the six measures (number of natural teeth, chewing ability, articulatory oral motor skills, tongue pressure, and subjective difficulties in eating and swallowing) as oral frailty, has widely utilized by scholars worldwide. OFI-8, also proposed by Tanaka et al. (3), defined oral frailty as the presence of four or more of the eight following items: eating tough food compared to 6 months ago, choking ability, using dentures, dry mouth, getting out frequency, eating hard food, brush teeth ≥3 times/day, and visiting a dental clinic at least annually.

Oral frailty, as a complex and multifaceted public health problem, can be caused by a lot of factors, including poor energy intake, psychological stress, and less social interaction, which leads to an increase in medical costs and is strictly associated with various health-related negative outcomes. Individuals with low chewing and swallowing ability may affect their intake of fiber, vitamins, folate, etc., which may lead to malnutrition, increase the risk of sarcopenia and frailty, and further reduce their oral function and quality of life (4, 5). Previous studies have also demonstrated that oral frailty was associated with malnutrition and increased risk of frailty, fall risk, sarcopenia, disability among community-dwelling older individuals (3, 6). However, it has been shown to be reversible through oral functional training for community-dwelling older individuals (7). A cluster-randomized controlled trial conducted by Shirobe et al. (8) found that a 12-week oral frailty measures program that includeded preparatory oral exercises, mouth-opening training, tongue pressure training, prosodic training, and masticatory training effectively alleviated oral frailty.

Therefore, researching the basic epidemiology of oral frailty in community-dwelling older individuals is paramount for healthcare professionals, policymakers, and clinicians.

At present, previous studies have shown that the prevalence of oral frailty among community-dwelling older adults varied greatly, ranging from 11% to 69% in different countries (9, 10). A systematic review conducted by Li et al. (11) reported that the overall prevalence of oral frailty among older people was 25%. However, the overall incidence of oral frailty among community-dwelling older adults has not been comprehensively summarized. Therefore, the objective of this meta-analysis and systematic review is to summarize the overall prevalence of oral frailty among community-dwelling older individuals and explore the relationship between study characteristics and the prevalence of oral frailty.

## Methods

This review was conducted following the Preferred Reporting Items for Systematic Reviews and Meta-analyses 2020 guidelines and registered in the International Prospective Register of Systematic Reviews (PROSPERO: CRD42024527800).

### Data sources and search strategies

#### Search strategy

We systematically searched seven databases, including three Chinese databases (China National Knowledge Infrastructure, Wangfang, Chinese Biological Medical Database) and four English databases (PubMed, EMBASE, Web of Science, The Cochrane Library), covering the period from January 1, 2017, to March 25, 2024. The search was limited to articles published in Chinese or English. The search method combined Medical Subject Headings (MeSH) and free terms, using the following search terms: “older adults” OR “geriatric” OR “senior” OR “aging” OR “old people” AND “oral health” OR “oral weakness” OR “oral frailty” OR “oral function” AND “community” OR “community-dwelling.” For a detailed search strategy, please refer to Supplemental File 1.

#### Eligibility criteria

Articles were included if they met the following criteria: (1) cross-sectional or cohort studies; (2) participants were older individuals aged 60 years or above, residing in the community; (3) oral frailty was assessed using OF-6 as developed by Tanaka et al., Oral Frailty Index-8 (OFI-8), and Oral Frailty Index-5 (OFI-5); and (4) reported prevalence of oral frailty.

Studies were excluded if they: (1) were randomized controlled studies; (2) were reviews, case reports, conference abstracts, or comments; (3) had an unclear definition of oral frailty; (4) included data from the same community participants; or (5) were duplicate publications or lacked original data.

#### Data extraction

Data from the recruited studies were extracted and cross-checked by two scholars (SW, YM). The extracted information included the first author's name, year of publication, country, study design, sample size, mean age, number of males/females, measurement methods, and the prevalence of oral frailty.

#### Quality assessment

The Agency for Healthcare Research and Quality (AHRQ) (12) tool was used to assess the quality of cross-sectional studies. The tool comprises 11 questions, where scores of 0–3, 4–7, and 8–11 denote low, moderate, and high quality, respectively. For cohort studies, the Newcastle-Ottawa Scale (NOS) (13), which covers three main domains (sample selection, comparability, and outcome assessment), was utilized to evaluate the risk of bias. The total score is 9 points, with scores of 0–4, 5–6, and 7–9 representing high, moderate, and low risk of bias, respectively.

#### Data analysis

The statistical software package Stata 14.0 was used to calculate the overall prevalence of oral frailty and corresponding 95% confidence intervals (CIs). The degree of heterogeneity was assessed using Cochrane's *Q, I*^2^, and *p*-value statistics, with *I*^2^ > 50% and a *p*-value < 0.05 considered indicative of high heterogeneity. A random-effects model was employed to summarize the pooled prevalence if eligible studies exhibited high heterogeneity; otherwise, a fixed-effects model was utilized. Subgroup analysis and meta-analysis were conducted to explore the sources of heterogeneity, while sensitivity analysis was performed to evaluate the reliability of the studies. Additionally, visual inspection of funnel plots and Egger's test were employed to assess publication bias. For all analyses, a *p*-value < 0.05 was considered statistically significant.

## Result

### Search results

**Figure 1:**
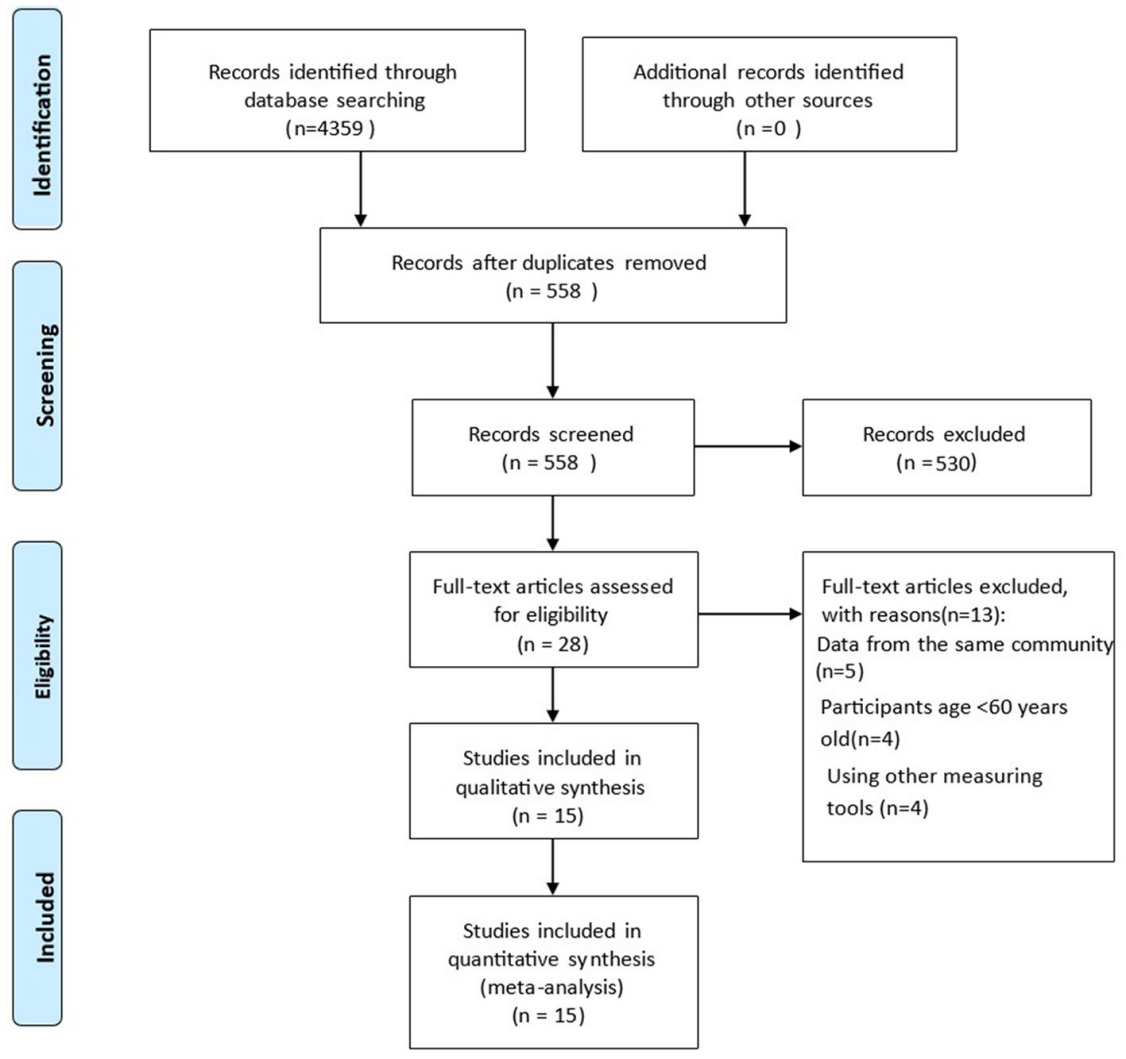
Flow diagram.

A total of 4,359 articles were identified through seven databases, of which 3,801 were duplicates. After screening titles and abstracts, 526 articles were removed. During full-text reading, 17 articles were excluded due to data duplication from the same community, participants under the age of 60 years, and the use of other measuring tools for oral frailty. Finally, 15 articles were included in this meta-analysis (Figure 1).

### Risk of bias

Thirteen articles were cross-sectional studies, with 9 studies considered to be of high quality and another 4 assessed as moderate quality. Additionally, 3 cohort studies were classified as having a low risk of bias (Table 1, Supplementary File 2).

**Table 1:** Characteristics of included studies.

**Author**	**Publication year**	**Country**	**Study design**	**Measurement**	**Mean age**	**Sample size**	**Prevalence (%)**			**Quality score**
							**Total**	**Man**	**Woman**	
Tanaka T (2)	2018	Japan	Cohort	OF-6	73.0 ± 5.5	2,011	15.8	15.7	16.0	7
Izutsu M (9)	2023	Japan	Cross-sectional	0FI-8	76.6 ± 5.8	238	10.5	15.7	8.3	8
Yin YH (10)	2024	China	Cross-sectional	OFI-8		310	69	70.7	67.5	9
Tang J (24)	2023	China	Cohort	OFI-8	72.7 ± 6.3	1,298	44.7	37.7	49.6	7
Wang L (25)	2023	China	Cross-sectional	OFI-8		223	59.2	63.1	55.8	6
Tu HJ (26)	2023	China	Cross-sectional	OFI-8	72.71 ± 8.0	204	33.8	24.6	46.4	6
Kuo YW (27)	2022	China	Cross-sectional	OFI-8	79.7 ± 7.2	308	60.4			8
Iwasaki M (28)	2021	Japan	Cross-sectional	OF-6	77.1 ± 4.7	1,082	21	22.1	20.2	8
Komatsu R (29)	2021	Japan	Cross-sectional	OF-6	72.8 ± 5.6	380	14.2	12.1	15.2	9
Yoshihiro K (30)	2020	Japan	Cross-sectional	OF-6	76.3 ± 6.5	679	22.5	22.3	22.7	9
Sanae H (31)	2020	Japan	Cross-sectional	OF-6	73.3 ± 6.6	682	9.5	7.5	10.8	9
Hoshino D (32)	2021	Japan	Cross-sectional	OF-6	75.9 ± 6.3	481	21.2			10
Song HZ (6)	2024	China	Cross-sectional	OFI-8	71.89 ± 7.58	409	41.3			7
Nishimoto M (33)	2023	Japan	Cohort	OF-6	72.2 ± 5.1	1,234	23.1	22.5	23.7	7
Iwasaki M (34)	2024	Japan	Cross-sectional	OFI-5	74.7 ± 5.5	1,206	36.7	36	38.8	8

### Description of included studies

A total of 10,745 community-dwelling older adults were included in this study, with 6 studies conducted in China and 9 articles conducted in Japan. The mean age of participants varied from 71.89 years old to 79.1 years old. Six studies used the OF-6 scale to measure oral frailty, 8 articles used the OFI-8 scale, and only 1 study used the OFI-5 scale. The sample sizes ranged from 204 to 2011 older individuals, with 6,020 females and 4,725 males. Furthermore, the prevalence of oral frailty ranged from 9.5% to 69.0%. Characteristics of the 15 eligible articles are shown in Table 1.

### Prevalence of oral frailty among community-dwelling older adults

**Figure 2:**
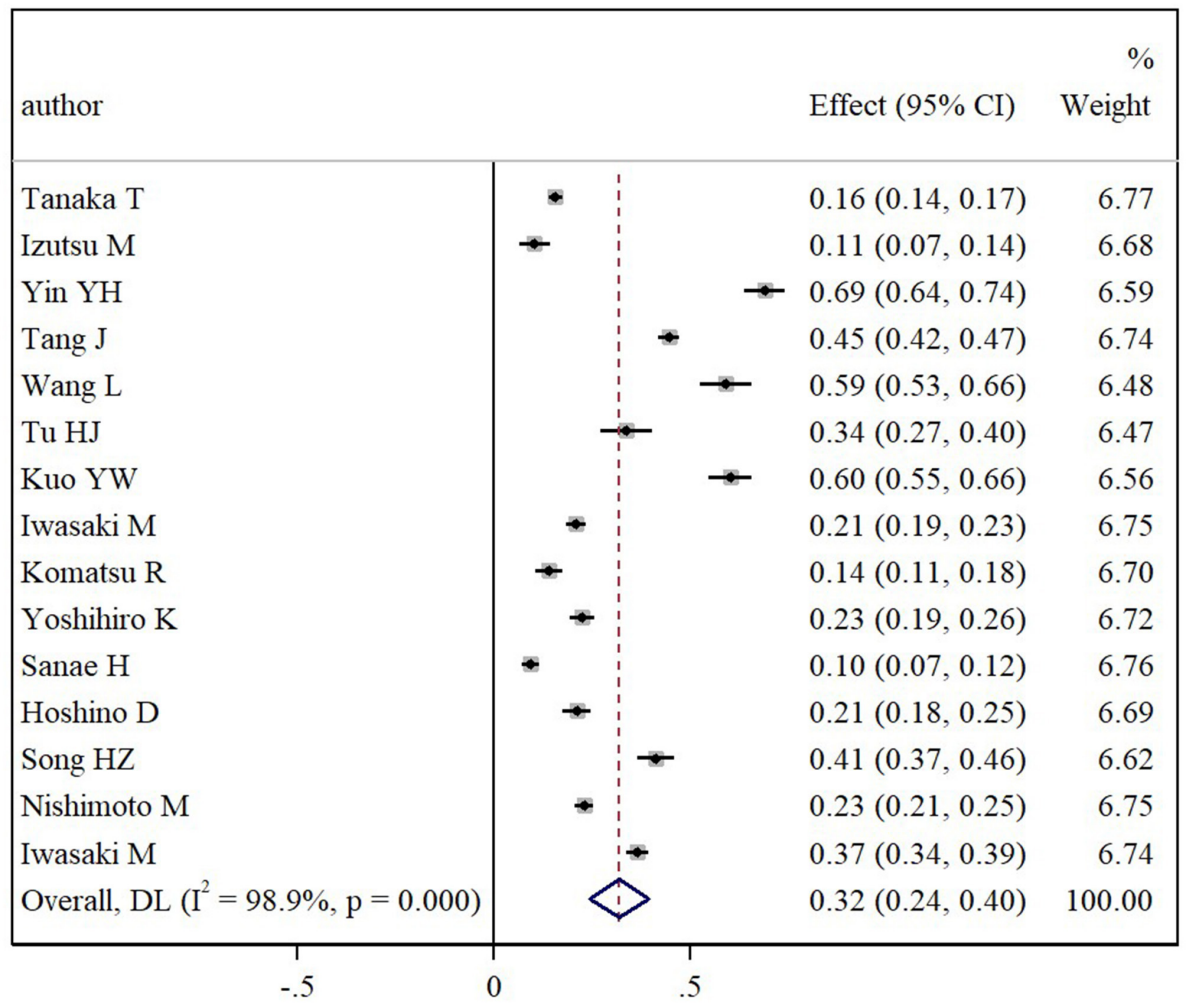
Foreast plot of pooled prevalence of oral frailty among community-dwelling older adults.

Twenty-one articles reported the prevalence estimates of oral frailty. The pooled prevalence of oral frailty among community-dwelling older adults was found to be 34% (95% CI: 27% to 41%), with considerable heterogeneity across all studies (*I*^2^ = 99.1%, *p* < 0.001) (Figure 2).

### Subgroup analysis and univariate meta-regression

Subgroup analysis could not fully elucidate the sources of heterogeneity between studies. However, studies conducted in different countries revealed that the pooled prevalence of oral frailty among community-dwelling older adults in China and Japan was 53% (95% CI: 42%−65%) and 22% (95% CI: 19%−39%), respectively. The pooled prevalence of oral frailty was 26% (95% CI: 16%−36%) for individuals with a mean age <74 years and 29% (95% CI: 18%-39%) for those aged ≥74 years. Cross-sectional studies yielded an oral frailty prevalence of 34% (95% CI: 25%−43%), while cohort studies reported a prevalence of 19% (95% CI: 12%−27%). Using the OFI-8 scale resulted in an oral frailty prevalence of 46% (95% CI: 31%−60%), whereas the prevalence was 18% (95% CI: 14%−22%) with the OF-6 scale, and 37% (95% CI: 34%−39%) with the OFI-5 scale. Furthermore, the pooled prevalence of oral frailty was 17% (95% CI: 13%−21%) for studies published before 2021 and 42% (95% CI: 31%−53%) for those published between 2022 and 2024. Studies with a sample size ≤ 500 had a prevalence of 39% (95% CI: 23%−54%), while those with a sample size >500 had a prevalence of 25% (95% CI: 16%−33%) (Table 2).

**Table 2:** Subgroup analysis.

**Subgroup**	**Studies**	**Prevalence**	**95%CI**	***P* value**	**Effect model**	***I*^2^ (%)**
**Country**						
China	5	53	0.42–0.65	<0.001	Random	96.4
Japan	10	22	0.19–0.39	<0.001	Random	97.6
**Mean age**						
<74	7	26	0.16–0.36	<0.001	Random	98.9
≥74	6	29	0.18–0.39	<0.001	Random	98.6
**Study design**						
Cross-sectional study	13	34	0.25–0.43	<0.001	Random	99.0
Cohort study	2	19	0.12–0.27	<0.001	Random	96.0
**Measurement**						
OFI-8	7	46	0.31–0.60	<0.001	Random	98.6
OFI-6	7	18	0.14–0.22	<0.001	Random	94.1
OFI-5	1	37	0.34–0.39	–	–	–
**Publishing year**						
Before 2021	6	17	0.13–0.21	<0.001	Random	93.3
2022–2024	9	42	0.31–0.53	<0.001	Random	98.7
**Sample size**						
≤ 500	8	39	0.23–0.54	<0.001	Random	98.9
>500	7	25	0.16–0.33	<0.001	Random	98.9

We selected covariates including country, mean age, study design, measurement, publishing year, and sample size for meta-regression. The results of univariate meta-regression showed that the pooled prevalence of oral frailty among community-dwelling older individuals was higher in studies conducted in China using the OFI-8 scale and published in 2022–2024. This suggests that country, measurement method, and publication year might contribute to model heterogeneity (Table 3).

**Table 3:** Univariate meta-regression.

**Factors**	***OR***	**95% CI**	**Adjust *R*^2^ (%)**	***P* value**
**Japan**	0.73	0.63–0.83	64.40	<0.001
**Cohort study**	0.86	0.63–1.18	0.04	0.332
**Measurement**			44.13	
OFI-5	0.92	0.66–1.28		0.579
OFI-6	0.76	0.64–0.90		0.004
**Publishing year**			38.99	
2022–2024	1.28	1.08–1.52		0.008
**Sample size**			7.32	
≤ 500	1.15	0.93–1.41		0.17

### Sensitivity analysis and publication bias

**Figure 3:**
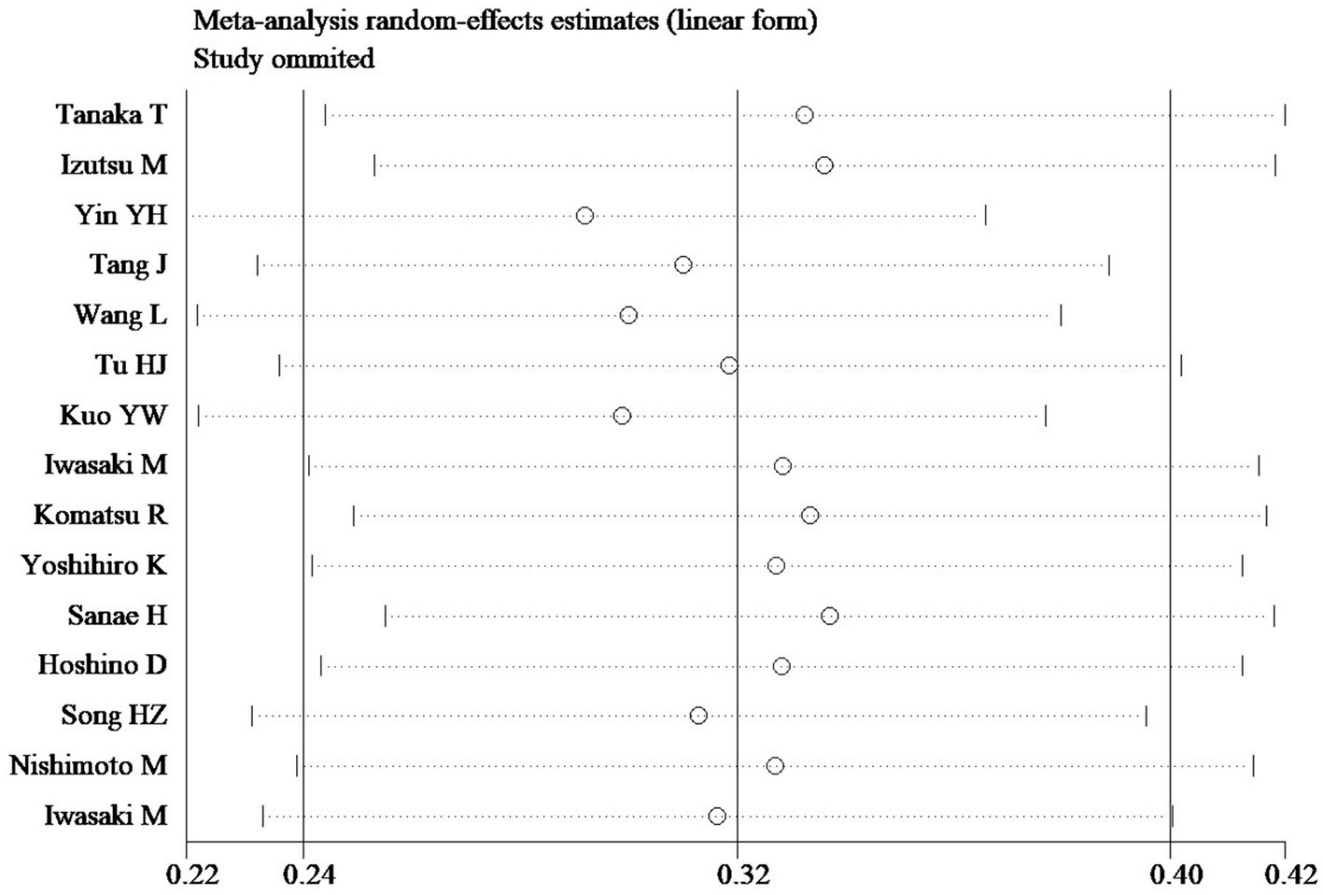
Sensitivity analysis.

**Figure 4:**
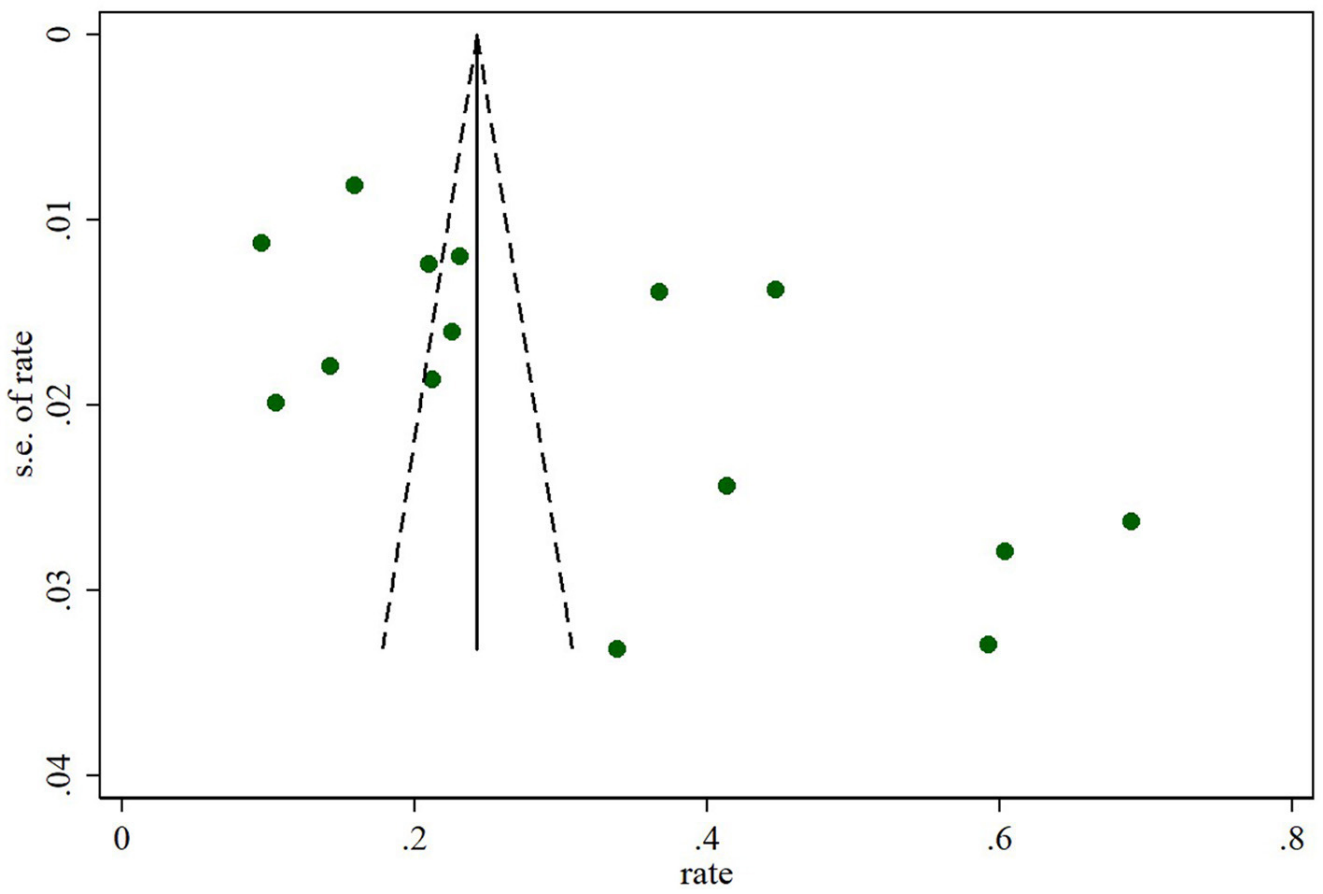
Funnel plot.

In sensitivity analyses, the overall prevalence of oral frailty among community-dwelling older adults remained stable, with no significant changes observed after removing each study (Figure 3). Furthermore, the funnel plot results and Egger's test (*t* = −0.36, *p* = 0.725) indicated no significant publication bias among the included studies (Figure 4).

## Discussion

Oral frailty, as an emerging concept, was developed and proposed in recent years. However, until now, researchers from other countries have conducted limited investigations into the oral frailty status among community-dwelling older adults, except for Japanese scholars. This study represents the most comprehensive systematic review and meta-analysis, summarizing the pooled prevalence of oral frailty among community-dwelling older individuals. The pooled prevalence of oral frailty among community-dwelling adults was found to be 34% (95% CI: 27%−41%), which was higher than the incidence rates of physical frailty (17.4%) (14), social frailty (20%) (15), and cognitive frailty (9%) (16) among community-dwelling older adults. The prevalence identified in this study suggests that oral frailty may become a significant concern in the future, emphasizing the importance for policymakers and healthcare professionals to implement effective measures for its prevention among community-dwelling older adults and to minimize its consequences. Given the considerable heterogeneity in this meta-analysis, univariate meta-regression was carried out to explore any potential covariates that might affect the overall prevalence estimate, and the results indicated that country and publishing year might influence the effect estimate.

Subgroup analysis revealed that the prevalence of oral frailty in China was significantly higher than in Japan. The possible reason for this discrepancy may be that developed countries like Japan have better oral health care services and technology (11), resulting in Japanese community-dwelling older people paying more attention to oral checkups. In the future, it is critical for developing countries like China to screen for oral frailty in community older adults further. Additionally, fewer studies have been conducted in China than in Japan, which may contribute to the possibility of bias due to small sample sizes. Thus, more correlational studies need to be explored.

The stratified analysis of mean age showed that the incidence of oral frailty in community-dwelling older individuals aged ≥74 years was higher than for those <74 years old. Previous studies have confirmed that with increasing age, the risk of physical frailty, sarcopenia, and other chronic diseases increases, all of which are associated with oral frailty (7). Additionally, as individuals age, they become more susceptible to experiencing malnutrition, chronic inflammation typical of oral disease, slight cognitive impairment, and depression, which may be underlying mechanisms affecting oral health and frailty (17–19).

The stratified analysis based on sample size indicated that the rate of oral frailty in small samples (≤ 500) was significantly higher than in large sample sizes (>500), consistent with other studies (20). Studies with smaller sample sizes often carry an elevated risk of publication bias and selection bias, potentially resulting in more extreme prevalence estimates and reduced statistical power to detect differences among groups (21, 22).

In our study, we found that the rate of oral frailty was notably higher when using the OFI-8 scale and OFI-5 scale compared to the OF-6 scale. Although both the OF-6 scale and OFI-8 scale were proposed by Tanaka et al. (2, 3), the assessment of oral functions (such as chewing ability and tongue pressure) in the OF-6 scale was conducted by trained dental hygienists with clinical experience, making it more complex to measure compared to the OFI-8 scale and OFI-5 scale (23). This complexity might have influenced the prevalence of oral frailty. However, a universally accepted measuring tool for oral frailty needs to be explored in the future.

Surprisingly, our results also found that the incidence of oral frailty published before 2021 was significantly lower than that published in 2022–2024. This difference may be explained by the use of different measuring tools, as the development of the OFI-8 scale and OFI-5 scale took place after 2021, consistent with our previous findings.

## Conclusion

In conclusion, this meta-analysis presents a comprehensive overview of the pooled prevalence of oral frailty among community-dwelling older adults and explores the relationship between various characteristics and the overall prevalence estimate. The findings reveal a pooled prevalence of oral frailty among community-dwelling older adults at 32% (95% CI: 25%−38%), with potential influences from different countries and publication years. These results provide robust evidence for researchers to delve deeper into understanding oral frailty and conduct further investigations. Moreover, the high prevalence of oral frailty among older adults in community settings underscores the urgency for policymakers and healthcare professionals to implement targeted preventive measures. Moving forward, future studies should prioritize high-quality research to confirm and build upon these findings.

## Limitations

This meta-analysis is subject to several limitations. Firstly, despite our efforts to account for potential sources of heterogeneity, such as country and publication year, we observed a high level of heterogeneity across the eligible studies, suggesting the presence of other unidentified factors contributing to this variation. Secondly, while we systematically searched seven electronic databases, the fact that all included studies originated from China and Japan limits the generalizability of our findings to a broader global context, as research from other regions, such as America and Europe, is limited in community-dwelling older adults. Lastly, the reliance on measuring tools for oral frailty primarily derived from Tanaka et al. introduces the possibility of measurement bias, warranting caution when interpreting the results of this study.

## Data Availability

The original contributions presented in the study are included in the article/Supplementary material, further inquiries can be directed to the corresponding author.

## References

[1] Japan Dental Association. Manual for oral frailty at dental clinics. (2019). Available online at https://www.ida.or.ip/dentist/oralflail/pdf/manual_all.pdf (accessed August 19, 2020).

[2] Tanaka T Takahashi K Hirano H Kikutani T Watanabe Y Ohara Y . Oral frailty as a risk factor for physical frailty and mortality in community-dwelling elderly. J Gerontol A Biol Sci Med Sci. (2018) 73:1661–7. 10.1093/gerona/glx225 29161342

[3] Tanaka T Hirano H Ohara Y Nishimoto M Iijima K. Oral frailty index-8 in the risk assessment of new-onset oral frailty and functional disability among community-dwelling older adults. (erratum in: *Arch Gerontol Geriatr*. 2021 sep–oct; 96:104466). Arch Gerontol Geriatr. (2021) 94:104340. 10.1016/j.archger.2021.104340 33529863

[4] de Sire A Ferrillo M Lippi L Agostini F de Sire R Ferrara PE . Sarcopenic dysphagia, malnutrition, and oral frailty in elderly: a comprehensive review. Nutrients. (2022) 14:982. 10.3390/nu14050982 35267957 PMC8912303

[5] Yoshida M Hiraoka A Takeda C Mori T Maruyama M Yoshikawa M . Oral hypofunction and its relation to frailty and sarcopenia in community-dwelling older people. Gerodontology. (2022) 39:26–32. 10.1111/ger.12603 34727388

[6] Song HZ Wei Y Wang Y Zhang J. The mediating effect of nutrition on oralfrailty and fall risk in community-dwelling elderly people. BMC Geriatr. (2024) 24:273. 10.1186/s12877-024-04889-3 38504156 PMC10953286

[7] Ohara Y Yoshida N Kono Y Hirano H Yoshida H Mataki S . Effectiveness of an oral health educational program on community-dwelling older people with xerostomia. Geriatr Gerontol Int. (2015) 15:481–9. 10.1111/ggi.12301 24796714

[8] Shirobe M Watanabe Y Tanaka T Hirano H Kikutani T Nakajo K . Effect of an oral frailty measures program on community-dwelling elderly people: a cluster-randomized controlled trial. Gerontology. (2022) 68:377–86. 10.1159/000516968 34247160 PMC9153353

[9] Izutsu M Hirayama K Su Y Yuki M. Risk factors for oral frailty among community-dwelling pre-frail older adults in Japan: a cross-sectional analysis. Community Dent Health. (2023) 40:221–6. 10.1922/CDH_00030Miku06 37988655

[10] Yin Y Zhao Y Fei Y Liu Y Ji Y Shan E . Epidemiology and risk factors of oral frailty among older people: an observational study from China. BMC Oral Health. (2024) 24:368. 10.1186/s12903-024-04149-1 38515048 PMC10958975

[11] Li T Shen Y Leng Y Zeng Y Li L Yang Z . The prevalence of oral frailty among older adults: a systematic review and meta-analysis. Eur Geriatr Med. (2024) 15:645–655. 10.1007/s41999-023-00930-7 38528284

[12] Zeng X Zhang Y Kwong JS Zhang C Li S Sun F . The methodological quality assessment tools for preclinical and clinical studies, systematic review and meta-analysis, and clinical practice guideline: a systematic review. J Evid Based Med. (2015) 8:2–10. 10.1111/jebm.12141 25594108

[13] Knapp G Hartung J. Improved tests for a random effects meta-regression with a single covariate. Stat Med. (2003) 22:2693–710. 10.1002/sim.1482 12939780

[14] Siriwardhana DD Hardoon S Rait G Weerasinghe MC Walters KR. Prevalence of frailty and prefrailty among community-dwelling older adults in low-income and middle-income countries: a systematic review and meta-analysis. BMJ Open. (2018) 8:e018195. 10.1136/bmjopen-2017-018195 29496895 PMC5855322

[15] Jia B Wang Z Zhang T Yue X Zhang S. Prevalence of social frailty and risk factors among community-dwelling older adults: a systematic review and meta-analysis. Arch Gerontol Geriatr. (2024) 123:105419. 10.1016/j.archger.2024.105419 38522381

[16] Qiu Y Li G Wang X Zheng L Wang C Wang C . Prevalence of cognitive frailty among community-dwelling older adults: a systematic review and meta-analysis. Int J Nurs Stud. (2022) 125:104112. 10.1016/j.ijnurstu.2021.104112 34758429

[17] Dibello V Lozupone M Manfredini D Dibello A Zupo R Sardone R . Oral frailty and neurodegeneration in Alzheimer's disease. Neural Regen Res. (2021) 16:2149–53. 10.4103/1673-5374.310672 33818486 PMC8354109

[18] Lin YC Huang SS Yen CW Kabasawa Y Lee CH Huang HL. Physical frailty and oral frailty associated with late-life depression in community-dwelling older adults. J Pers Med. (2022) 12:459. 10.3390/jpm12030459 35330459 PMC8954826

[19] Hu S Li X. An analysis of influencing factors of oral frailty in the elderly in the community. BMC Oral Health. (2024) 24:260. 10.1186/s12903-024-03946-y 38383363 PMC10882750

[20] Dou J Liu H Ma Y Wu YY Tao XB. Prevalence of post-dialysis fatigue: a systematic review and meta-analysis. BMJ Open. (2023) 13:e064174. 10.1136/bmjopen-2022-064174 37311633 PMC10277119

[21] Liu J Gan Y Jiang H Li L Dwyer R Lu K . Prevalence of workplace violence against healthcare workers: a systematic review and meta-analysis. Occup Environ Med. (2019) 76:927–37. 10.1136/oemed-2019-105849 31611310

[22] Nayak BK. Understanding the relevance of sample size calculation. Indian J Ophthalmol. (2010) 58:469–70. 10.4103/0301-4738.71673 20952828 PMC2993974

[23] Minakuchi S Tsuga K Ikebe K Ueda T Tamura F Nagao K . Oral hypofunction in the older population: Position paper of the Japanese Society of Gerodontology in 2016. Gerodontology. (2018) 35:317–324. 10.1111/ger.12347 29882364

[24] Tang J Tang XY Zeng L Chen H Yang X Zhou QX . Prevalence and influencing factors of oral frailty in the elderly of rural areas in Guizhou Province. Chin J Prev Con Chron Dis. (2023) 31:327–31. 10.16386/j.cjpccd.issn.1004-6194.2023.05.002

[25] Wang L Cui M Wang T Wang E Pu XL Jia XY . Oral frailty riskarid its infuencing factors in community-dwelling elderly population. J Nurs Sci. (2023) 38:112–6. 10.3870/j.issn.1001-4152.2023.18.112

[26] Tu HJ Zhang SY Fang YH He GJ. Current situation and influencing factors of oral frailty in the community elderly. Chin J Nurs. (2023) 58:1351–6. 10.3761/j.issn.0254-1769.2023.11.011

[27] Kuo YW Lee JD. Association between oral frailty and physical frailty among rural middle-old community-dwelling people with cognitive decline in Taiwan: a cross-sectional study. Int J Environ Res Public Health. (2022) 19:2884. 10.3390/ijerph19052884 35270577 PMC8909940

[28] Iwasaki M Watanabe Y Motokawa K Shirobe M Inagaki H Motohashi Y . Oral frailty and gait performance in community-dwelling older adults: findings from the Takashimadaira study. J Prosthodont Res. (2021) 65:467–73. 10.2186/jpr.JPR_D_20_00129 33612666

[29] Komatsu R Nagai K Hasegawa Y Okuda K Okinaka Y Wada Y . association between physical frailty subdomains and oral frailty in community-dwelling older adults. Int J Environ Res Public Health. (2021) 18:2931. 10.3390/ijerph18062931 33809322 PMC8001836

[30] Kugimiya Y Watanabe Y Ueda T Motokawa K Shirobe M Igarashi K . Rate of oral frailty and oral hypofunction in rural community-dwelling older Japanese individuals. Gerodontology. (2020) 37:342–52. 10.1111/ger.12468 32141117

[31] Hironaka S Kugimiya Y Watanabe Y Motokawa K Hirano H Kawai H . Association between oral, social, and physical frailty in community-dwelling older adults. Arch Gerontol Geriatr. (2020) 89:104105. 10.1016/j.archger.2020.104105 32480111

[32] HoshinoD HiranoH EdahiroA MotokawaK ShirobeM WatanabeY . Association between oral frailty and dietary variety among community-dwelling older persons: a cross-sectional study. J Nutr Health Aging. (2021) 25:361–8. 10.1007/s12603-020-1538-633575729

[33] Nishimoto M Tanaka T Hirano H Watanabe Y Ohara Y Shirobe M . Severe Periodontitis increases the risk of oral frailty: a six-year follow-up study from Kashiwa cohort study. Geriatrics. (2023) 8:25. 10.3390/geriatrics8010025 36826367 PMC9956982

[34] Iwasaki M Shirobe M Motokawa K Tanaka T Ikebe K Ueda T . Prevalence of oral frailty and its association with dietary variety, social engagement, and physical frailty: Results from the Oral Frailty 5-Item Checklist. Geriatr Gerontol Int. (2024) 24:371–7. 10.1111/ggi.14846 38390632

