# Supplementary material for: Prevalence of oral frailty in community-dwelling older adults: a systematic review and meta-analysis
Source: Front Public Health. 2025 May 1;13:1423387. doi: 10.3389/fpubh.2025.1423387 (PMC12078140; doi:10.3389/fpubh.2025.1423387)
Supplement: Supplementary file 1 [file Supplementary_file_1.docx]

PubMed:

‘Aging’ [Mesh] OR ‘elderly’[Title/Abstract] OR ‘older adult*’[Title/Abstract] OR ‘geriatric’[Title/Abstract] OR ‘senior’ [Title/Abstract] OR ‘old people’ [Title/Abstract] AND ‘Oral health’ Title/Abstract] OR ‘Oral weakness’ [Title/Abstract] OR ‘Oral frailty’ [Title/Abstract] OR ‘Oral function’ [Title/Abstract] AND ‘Community’ [Title/Abstract] OR ‘Community-dwelling’ [Title/Abstract]

Web of Science

**TS= (Aging OR elderly OR older adult* OR geriatric OR senior OR old people) AND TS= (Oral health OR Oral weakness OR Oral frailty OR Oral function) AND TS= (Community OR Community-dwelling)**

EMBASE

‘Aging’/exp OR ‘elderly’:ab ti OR ‘older adult*’:ab ti OR ‘geriatric’: ab ti OR ‘senior’:ab ti OR ‘old people’: ab ti AND ‘Oral health’ : ab ti OR ‘oral weakness’: ab ti OR ‘oral frailty’: ab ti OR ‘oral function’: ab ti AND ‘community’:ab ti OR ‘community-dwelling’: ab ti

Cochrane Library

MeSH descriptor: [Aged] in all MeSH products OR (elderly):ti,ab,kw OR (older adult* ):ti,ab,kw OR (geriatric):ti,ab,kw OR (senior):ti, ab,kw OR (old people);ti, ab,kw AND MeSH descriptor: [Oral Health] explode all trees OR (Oral weakness):ti,ab,kw OR (Oral frailty):ti,ab,kw OR (Oral function):ti,ab,kw AND (community):ti,ab,kw OR (community-dwelling):ti,ab,kw

Wangfang/CNK/CBM

(AB=older people OR old adults OR aging) AND (oral frailty)
