# Supplementary material for: Prevalence of oral frailty in community-dwelling older adults: a systematic review and meta-analysis
Source: Front Public Health. 2025 May 1;13:1423387. doi: 10.3389/fpubh.2025.1423387 (PMC12078140; doi:10.3389/fpubh.2025.1423387)
Supplement: Supplementary file 2 [file Supplementary_file_2.docx]

1.1 Quality assessment for cross-sectional studies

**ARHQ Methodology Checklist Cross-sectional/Prevalence Study**

| **Item** | **Yes** | **No** | **Unclear** |
| --- | --- | --- | --- |
| ①Define the source of information(survey,record,review) |  |  |  |
| ②List inclusion and exclusion criteria for unexposed subjects(case an controls) or fefer to previous publications |  |  |  |
| ③Indicate time period used for identifying patients |  |  |  |
| ④Indicate whether subjects were consecutive if not population-based |  |  |  |
| ⑤Indicate if evaluators of subjective components of study were blind to other aspects of the status of the participants |  |  |  |
| ⑥Describe any assessment undertaken for quality assureance purpose(e.g,retest of primary outcome measurement) |  |  |  |
| ⑦Explain any exclusions of data from analysis |  |  |  |
| ⑧Describe howconfounding was assessed and-or controlled |  |  |  |
| ⑨If applicable, explain how missing data were handled in the analysis |  |  |  |
| ⑩Summarize patient response rates and completenss of data collection |  |  |  |
| ⑪Clarify what follow up,if any,was expected and the percentage of patients for which incomplete data or follow-up was obtained |  |  |  |

**Resule of qualitity of assessment using ARHQ for cross-sectional studies**

| Author | Item1 | Item2 | Item3 | Item4 | Item5 | Item6 | Item7 | Item8 | Item9 | Item10 | Item11 |
| --- | --- | --- | --- | --- | --- | --- | --- | --- | --- | --- | --- |
| Izutsu M | Yes | Yes | Yes | Yes | Unclear | Unclear | Yes | No | Yes | Yes | Yes |
| Yin YH | Yes | Yes | Yes | Yes | Unclear | Unclear | Yes | Yes | Yes | Yes | Yes |
| Wang L | Yes | Yes | Yes | Yes | Unclear | Unclear | No | No | Yes | Yes | Unclear |
| Tu HJ | Yes | Yes | Yes | Yes | Unclear | Unclear | No | No | Yes | Yes | Unclear |
| Kuo YW | Yes | Yes | Yes | Yes | Unclear | Unclear | Yes | No | Yes | Yes | Yes |
| Iwasaki M | Yes | Yes | Yes | Yes | Unclear | Unclear | Yes | No | Yes | Yes | Yes |
| Komatsu R | Yes | Yes | Yes | Yes | Unclear | Unclear | Yes | Yes | Yes | Yes | Yes |
| Yoshihiro K | Yes | Yes | Yes | Yes | Unclear | Yes | Yes | Unclear | Yes | Yes | Yes |
| Sanae H | Yes | Yes | Yes | Yes | Unclear | Yes | Yes | Unclear | Yes | Yes | Yes |
| Hoshino D | Yes | Yes | Yes | Yes | Unclear | Yes | Yes | Yes | Yes | Yes | Yes |
| Song HZ | Yes | Yes | Yes | Yes | Unclear | Unclear | Yes | Unclear | Yes | Yes | Unclear |
| Iwasaki M | Yes | Yes | Yes | Yes | Unclear | Unclear | Yes | No | Yes | Yes | Yes |

1.2 Quality assessment for cohort studies

**The Nwecastle-Ottawa Quality Assessment Scale(NOS)**

| Selection | ①Is the case definition adequate | yes, with independent validation |
| --- | --- | --- |
|  |  | yes, eg record linkage or based on self reports |
|  |  | no description |
|  |  |  |
|  | ②Representativeness of the cases | consecutive or obviously representative series of cases |
|  |  | potential for selection biases or not stated |
|  |  |  |
|  | ③Selection of Controls | community controls |
|  |  | hospital controls |
|  |  | no description |
|  |  |  |
|  | ④Definition of Controls | no history ofdisease (endpoint) |
|  |  | no description of source |
|  |  |  |
| Comparability | ①Comparability of cases and controls on the basis of the design or analysis | study controls for the most important factor |
|  |  | study controls for any additional factor |
|  |  |  |
| Exposure | ①Ascertainment of exposure | secure record |
|  |  | structured interview where blind to case/control status |
|  |  | interview not blinded to case/control status |
|  |  | written self report or medical record only |
|  |  | no description |
|  |  |  |
|  | ②Same method of ascertainment for cases and controls | Yes |
|  |  | No |
|  |  |  |
|  | ③Non-Response rate | same rate for both groups |
|  |  | non respondents described |
|  |  | rate diferent and no designation |

**Resule of qualitity of assessment using NOS for cohort studies**

| **Author** | **Selection** | | | | **Comparability** | **Exposure** | | |
| --- | --- | --- | --- | --- | --- | --- | --- | --- |
|  | **①** | **②** | **③** | **④** | **①** | **①** | **②** | **③** |
| Tanaka T | **1** | **1** | **1** | **1** | **0** | **1** | **1** | **1** |
| Tang J | **1** | **1** | **1** | **1** | **0** | **1** | **1** | **1** |
| Nishimoto M | **1** | **1** | **1** | **1** | **0** | **1** | **1** | **1** |
